# Evaluation of Environmental Stability and Disinfectant Effectiveness for Human Coronavirus OC43 on Human Skin Surface

**DOI:** 10.1128/spectrum.02381-22

**Published:** 2023-02-22

**Authors:** Naoto Watanabe, Ryohei Hirose, Katsuma Yamauchi, Hajime Miyazaki, Risa Bandou, Takuma Yoshida, Toshifumi Doi, Ken Inoue, Osamu Dohi, Naohisa Yoshida, Kazuhiko Uchiyama, Takeshi Ishikawa, Tomohisa Takagi, Hideyuki Konishi, Hiroshi Ikegaya, Takaaki Nakaya, Yoshito Itoh

**Affiliations:** a Molecular Gastroenterology and Hepatology, Graduate School of Medical Science, Kyoto Prefectural University of Medicine, Kyoto, Japan; b Department of Infectious Diseases, Graduate School of Medical Science, Kyoto Prefectural University of Medicine, Kyoto, Japan; c Department of Forensics Medicine, Graduate School of Medical Science, Kyoto Prefectural University of Medicine, Kyoto, Japan; University of Georgia

**Keywords:** HCoV-OC43, environmental stability, virus survival, disinfection effectiveness

## Abstract

The environmental stability of human coronavirus OC43 (HCoV-OC43) on the surface of human skin and the effectiveness of disinfectant against HCoV-OC43, which are important to prevent contact transmission, have not been clarified in previous studies. Using previously generated models, we evaluated HCoV-OC43 stability and disinfection effectiveness. Then we compared the results with those for severe acute respiratory syndrome coronavirus 2 (SARS-CoV-2). The median survival time of HCoV-OC43 on the surface of human skin was 24.6 h (95% confidence interval, 19.7 to 29.6 h), which was higher than that of SARS-CoV-2 (10.8 h). Although the *in vitro* disinfectant effectiveness evaluation showed that HCoV-OC43 has a higher ethanol resistance than SARS-CoV-2, HCoV-OC43 on the skin surface was completely inactivated by a minimum of 50% ethanol within 5 s (the log reduction values were >4.0). Moreover, 1.0% chlorhexidine gluconate and 0.2% benzalkonium chloride showed relatively high disinfectant effectiveness, and the log reduction values when these disinfectants were applied for 15 s were >3.0. HCoV-OC43 is highly stable on the skin surface, which may increase the risk of contact transmission. Although HCoV-OC43 has relatively high ethanol resistance, appropriate hand hygiene practices with current alcohol-based disinfectants sufficiently reduce the risk of contact transmission.

**IMPORTANCE** This study revealed the environmental stability of HCoV-OC43 and disinfectant effectiveness against HCoV-OC43, which had not been demonstrated in previous studies. HCoV-OC43 is highly stable on the surface of human skin, with a survival time of approximately 25 h. High stability of HCoV-OC43 may increase the risk of contact transmission. Furthermore, the *in vitro* disinfectant effectiveness evaluation showed that HCoV-OC43, which is classified as an envelope virus, has a relatively high ethanol resistance. This finding suggests that disinfectant effectiveness may vary greatly depending on the virus and that each virus targeted for infection control should be evaluated individually. HCoV-OC43 on the skin surface was rapidly inactivated by 50% ethanol, which suggests that appropriate hand hygiene practices with current alcohol-based disinfectants can sufficiently reduce the risk of HCoV-OC43 contact transmission.

## INTRODUCTION

Human coronavirus (HCoV) causes upper and lower respiratory tract infections (1), and HCoV is a prevalent cause of colds worldwide (2–4). While immunocompetent adults infected with HCoV develop asymptomatic or mild upper respiratory tract inflammation and often fully recover, children, the elderly, and immunocompromised individuals may develop severe lower respiratory tract inflammation (i.e., pneumonia), which may result in death (1, 5, 6). Four types of HCoV have been identified: HCoV-229E, HCoV-OC43, HCoV-NL63, and HCoV-HKU1. HCoV-229E and HCoV-NL63 are classified in the *Alphacoronavirus* genus, while HCoV-OC43 and HCoV-HKU1 are classified in the *Betacoronavirus* genus (7, 8). Middle east respiratory syndrome coronavirus (MERS-CoV) and severe acute respiratory syndrome coronavirus 1 (SARS-CoV-1), previously prevalent in some areas, and SARS-CoV-2, the current source of the worldwide pandemic, cause severe lower respiratory tract infections with high mortality rates. These viruses are also classified in the *Betacoronavirus* genus (7). Therefore, HCoV-OC43 has been studied as a surrogate for SARS-CoV-2 (9–12).

Contact and droplet transmission are the primary transmission pathways for HCoV-OC43 (13, 14), and hand hygiene is important for prevention. The viral stability and disinfectant effectiveness against HCoV have been reported in several studies on HCoV-229E (15–18). Since the conventional method for titrating HCoV-OC43 (using the indirect immunoperoxidase assay [19–24]) was cumbersome, the stability of HCoV-OC43 and the efficacy of disinfectants against HCoV-OC43 have scarcely been evaluated (18, 25). Additionally, the stability on human skin and the disinfectant effectiveness against all HCoVs have not been evaluated, although this information is very important for disease prevention. Ethanol (EA), isopropanol (IPA), chlorhexidine gluconate (CHG), benzalkonium chloride (BAC), and povidone-iodine (PVP-I) are commonly used on human skin for hand hygiene, and these were evaluated in this study.

For this study, we established a model to evaluate viral stability and disinfection effectiveness against viruses on human skin obtained from forensic autopsy specimens in our previous studies (26, 27). Additionally, we developed a simple and accurate 50% tissue culture infectious dose (TCID_50_) assay by cytopathic effect (CPE) observation with HCoV-OC43 infecting VeroE6 cells expressing transmembrane protease serine 2 (TMPRSS2) (28). These models were used to evaluate the stability of HCoV-OC43 and disinfection effectiveness of various disinfectants against HCoV-OC43 on human skin.

## RESULTS

### Evaluation of HCoV-OC43 stability on the surface of human skin.

**FIG 1:**
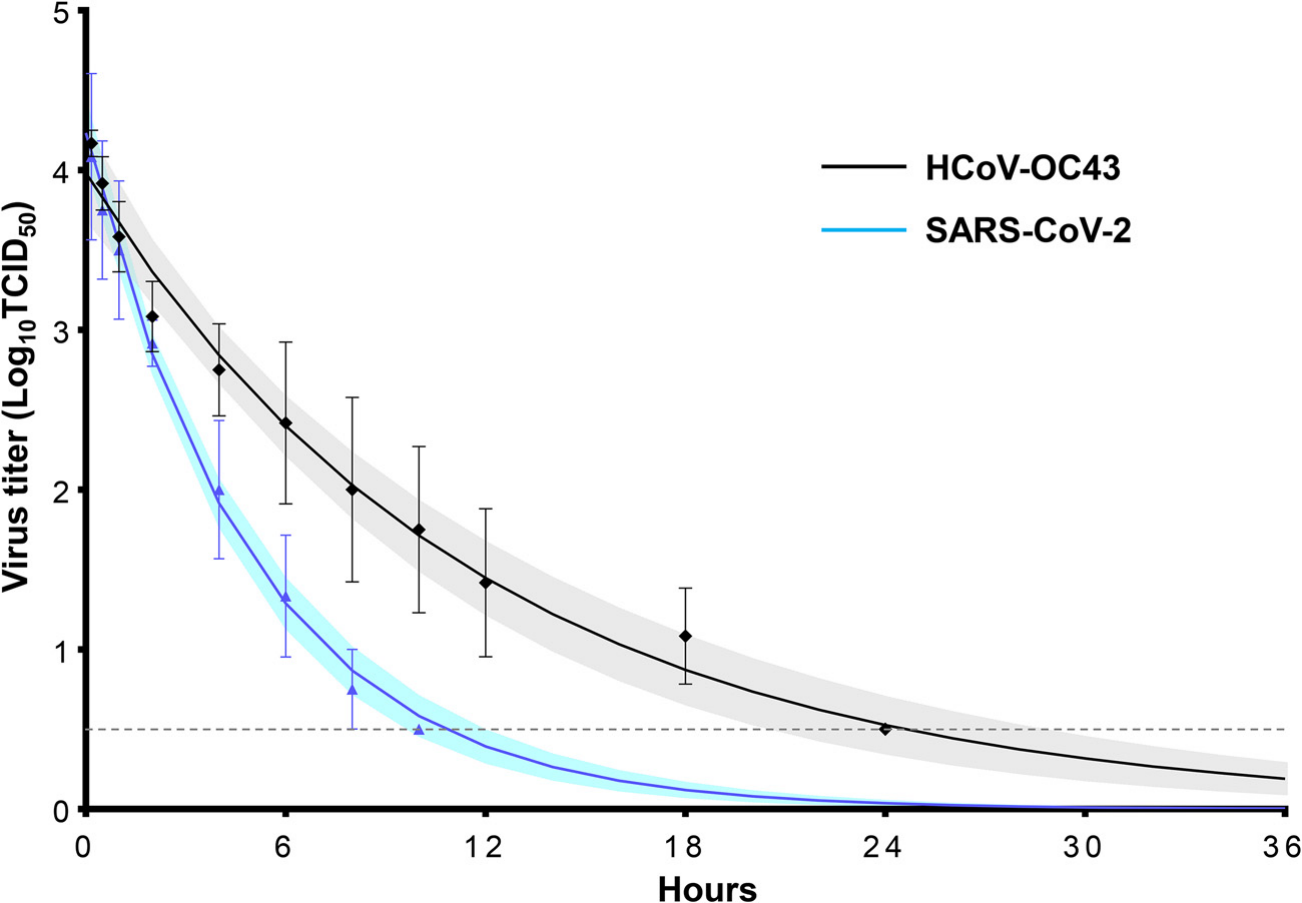
Environmental stability of HCoV-OC43 and SARS-CoV-2 on the surface of human skin. The elapsed time was defined as an explanatory variable (*x* axis), and the log virus titer was defined as an explained variable (*y* axis). Least-squares linear-regression analysis was performed with a logarithmic link function for each virus to generate a regression curve. The upper and lower confidence limits are represented by shaded curves. The dashed horizontal line represents the detection limit titer. The data are expressed as the means ± standard errors of the means for three independent experiments.

HCoV-OC43 displayed a significantly longer survival time than SARS-CoV-2. The survival time of HCoV-OC43 on the skin was 24.59 h (95% confidence interval [CI], 19.71 to 29.62), whereas that of SARS-CoV-2 was 10.78 h (95% CI, 9.32 to 12.25) (Fig. 1 and Table 1). The half-lives of HCoV-OC43 were 1.19 h (95% CI, 0.95 to 1.52) and 1.78 h (95% CI, 1.42 to 2.28) when the residual viral titers on the skin were 10^3^ TCID_50_ and 10^2^ TCID_50_, respectively. The half-lives of SARS-CoV-2 were 0.51 h (95% CI, 0.44 to 0.59) and 0.76 h (95% CI, 0.65 to 0.89) when the residual viral titers on the skin were 10^3^ TCID_50_ and 10^2^ TCID_50_, respectively. Overall, HCoV-OC43 had a significantly longer half-life than SARS-CoV-2.

**TABLE 1:** Survival time and half-life of HCoV-OC43 and SARS-CoV-2 on the human skin surface

Virus	Survival time, h, median (95% CI)	Half-life, h, median (95% CI)	
		3 log_10_ TCID_50_s	2 log_10_ TCID_50_s
HCoV-OC43	24.59 (19.71–29.62)	1.19 (0.95–1.52)	1.78 (1.42–2.28)
SARS-CoV-2	10.78 (9.32–12.25)	0.51 (0.44–0.59)	0.76 (0.65–0.89)

### *In vitro* evaluation of disinfectant effectiveness.

The disinfectant effectiveness against HCoV-OC43 was evaluated *in vitro* (Table 2; see also Fig. S1 in the supplemental material). HCoV-OC43 was rapidly (within 5 s) and completely inactivated with 60% EA, 80% EA, and 70% IPA. However, HCoV-OC43 could not be effectively inactivated with less than 50% EA. Specifically, the log reduction values after the reaction of 50% EA for 5, 15, and 60 s were 2.33 ± 0.77, 3.83 ± 0.31, and 4.08 ± 0.31, respectively. The log reduction values after reaction with 40% EA for 5, 15, and 60 s were 1.50 ± 0.35, 2.17 ± 0.12, and 2.75 ± 0.54, respectively. The log reduction values after reaction with 20% EA for 5, 15, and 60 s were 0.00 ± 0.20, 0.42 ± 0.12, and 0.75 ± 0.35, respectively.

**TABLE 2:** *In vitro* evaluation of disinfectant effectiveness against HCoV-OC43^*a*^

Disinfectant	Log reduction (mean ± SE)		
	5 s	15 s	60 s
80% EA	4.17 ± 0.24	4.17 ± 0.24	4.17 ± 0.24
60% EA	4.17 ± 0.24	4.17 ± 0.24	4.17 ± 0.24
50% EA	2.33 ± 0.77	3.83 ± 0.31	4.08 ± 0.31
40% EA	1.50 ± 0.35	2.17 ± 0.12	2.75 ± 0.54
20% EA	0.00 ± 0.20	0.42 ± 0.12	0.75 ± 0.35
70% IPA	4.17 ± 0.24	4.17 ± 0.24	4.17 ± 0.24
0.2% CHG	0.83 ± 0.31	1.00 ± 0.71	1.08 ± 0.12
1.0% CHG	1.42 ± 0.42	1.50 ± 0.35	1.75 ± 0.00
0.05% BAC	1.17 ± 0.24	1.42 ± 0.31	2.00 ± 0.61
0.2% BAC	2.33 ± 0.42	3.42 ± 0.42	4.08 ± 0.12
10% PVP-I	4.17 ± 0.24	4.17 ± 0.24	4.17 ± 0.24

a EA, ethanol; IPA, isopropanol; CHG, chlorhexidine gluconate; BAC, benzalkonium chloride; PVP-I, povidone iodine; HCoV-OC43, human coronavirus OC43. The log reduction values were calculated to evaluate the disinfectant effectiveness under each condition.

CHG, BAC, and PVP-I were also tested for disinfectant effectiveness against HCoV-OC43. The log reduction values after reaction with 0.2% CHG for 5, 15, and 60 s were 0.83 ± 0.31, 1.00 ± 0.71, and 1.08 ± 0.12, respectively. The log reduction values after reaction with 1.0% CHG for 5, 15, and 60 s were 1.42 ± 0.42, 1.50 ± 0.35, and 1.75 ± 0.00, respectively. The disinfectant effectiveness of CHG increased with increasing concentrations and reaction times but was significantly lower than those of 60% EA, 80% EA, and 70% IPA (*P < *0.005). The log reduction values after reaction with 0.05% BAC for 5, 15, and 60 s were 1.17 ± 0.24, 1.42 ± 0.31, and 2.00 ± 0.61, respectively. The log reduction values after reaction with 0.2% BAC for 5, 15, and 60 s were 2.33 ± 0.42, 3.42 ± 0.42, and 4.08 ± 0.12, respectively. The disinfectant effectiveness of BAC also increased with increasing concentrations and reaction times. Interestingly, 0.2% BAC showed a relatively strong disinfectant effectiveness against HCoV-OC43; it was rapidly and completely inactivated within 5 s using 10% PVP-I, similar to the case with 60 and 80% EA and 70% IPA.

**FIG 2:**
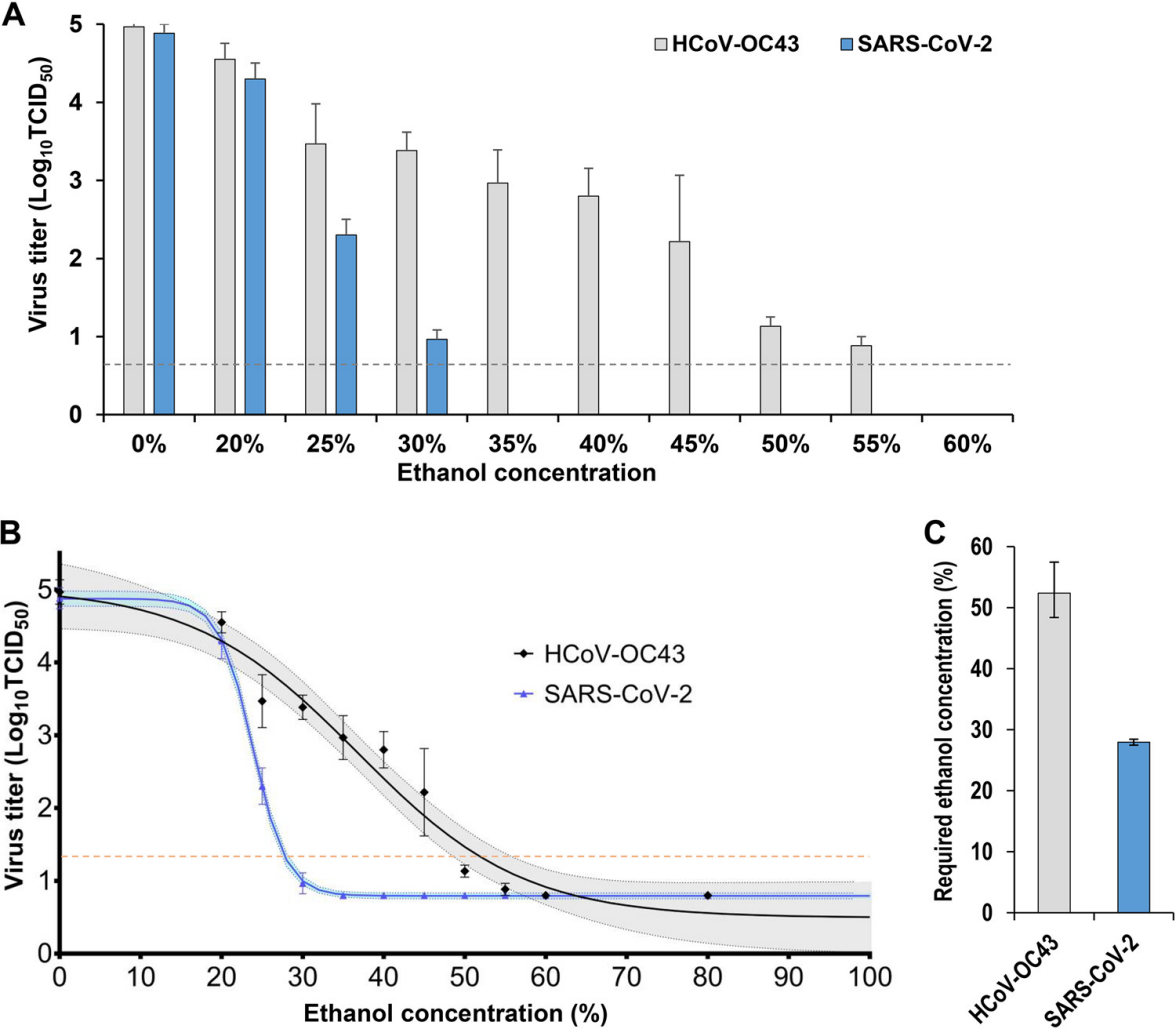
Analysis of disinfectant effectiveness of various concentrations of ethanol against HCoV-OC43 and SARS-CoV-2 (*in vitro* evaluation). (A) Residual viral titers were measured after reaction with ethanol at concentrations of 20, 25, 30, 35, 40, 45, 50, 55, 60, and 80% for 15 s. The bars below the detection sensitivity are omitted. Three independent experiments were performed, and the results of the log virus titer are expressed as mean ± standard errors of the means. The dashed horizontal line represents the detection limit titer. (B) The ethanol concentration was defined as an explanatory variable (*x* axis), and the log virus titer concentration was defined as an explained variable (*y* axis). Nonlinear regression analyses were conducted using a four-parameter logistic model. The upper and lower confidence limits are represented by dotted curves. (C) The ethanol concentration required to achieve a logarithmic decrease of 3.5 in viral titer concentration in a 15-s disinfection reaction time (notated as required ethanol concentration) was defined as the *x* value when the *y* value of the regression curve was 1.3.

Moreover, the ethanol concentration that was required to achieve a logarithmic decrease of 3.5 in virus titer concentration in a 15-s disinfection reaction (defined as required EA concentration) was calculated. The required EA concentrations for HCoV-OC43 and SARS-CoV-2 were 52.39% (95% CI, 48.38 to 57.47) and 27.94% (95% CI, 27.45 to 28.44), respectively. These values showed that HCoV-OC43 had significantly higher EA resistance than SARS-CoV-2 (Fig. 2).

### Evaluation of disinfectant effectiveness against HCoV-OC43 on the human skin surface.

The disinfectant effectiveness against HCoV-OC43 was evaluated on the surface of human skin (Table 3 and Fig. S1). HCoV-OC43 was rapidly (within 5 s) and completely inactivated with 50, 60, and 80% EA and 70% IPA. However, HCoV-OC43 was not effectively inactivated by less than 40% EA. Specifically, the log reduction values after reaction with 40% EA for 5, 15, and 60 s were 3.42 ± 0.47, 4.00 ± 0.20, and 4.08 ± 0.12, respectively. The log reduction values after reaction with 20% EA for 5, 15, and 60 s were 1.00 ± 0.41, 1.50 ± 0.20, and 2.42 ± 0.24, respectively.

**TABLE 3:** Evaluation of disinfectant effectiveness against HCoV-OC43 on the surface of human skin

Disinfectant	Log reduction (mean ± SE)^*a*^		
	5 s	15 s	60 s
80% EA	4.17 ± 0.12	4.17 ± 0.12	4.17 ± 0.12
60% EA	4.17 ± 0.12	4.17 ± 0.12	4.17 ± 0.12
50% EA	4.17 ± 0.12	4.17 ± 0.12	4.17 ± 0.12
40% EA	3.42 ± 0.47	4.00 ± 0.20	4.08 ± 0.12
20% EA	1.00 ± 0.41	1.50 ± 0.20	2.42 ± 0.24
70% IPA	4.17 ± 0.12	4.17 ± 0.12	4.17 ± 0.12
0.2% CHG	1.50 ± 0.61	1.58 ± 0.12	1.83 ± 0.42
1.0% CHG	2.25 ± 0.41	3.08 ± 0.24	3.33 ± 0.47
0.05% BAC	1.75 ± 0.54	2.08 ± 0.12	2.83 ± 0.66
0.2% BAC	2.92 ± 0.62	3.83 ± 0.24	4.17 ± 0.12
10% PVP-I	4.17 ± 0.12	4.17 ± 0.12	4.17 ± 0.12

a The log reduction values were calculated to evaluate the disinfectant effectiveness under each condition.

The log reduction values after reaction with 0.2% CHG for 5, 15, and 60 s were 1.50 ± 0.61, 1.58 ± 0.12, and 1.83 ± 0.42, respectively. The log reduction values after reaction with 1.0% CHG for 5, 15, and 60 s were 2.25 ± 0.41, 3.08 ± 0.24, and 3.33 ± 0.47, respectively. The disinfectant effectiveness of CHG increased with increasing concentrations and reaction times, and 1.0% CHG displayed a relatively strong disinfectant effectiveness against HCoV-OC43. The log reduction values after reaction with 0.05% BAC for 5, 15, and 60 s were 1.75 ± 0.54, 2.08 ± 0.12, and 2.83 ± 0.66, respectively. The log reduction values after reaction with 0.2% BAC for 5, 15, and 60 s were 2.92 ± 0.62, 3.83 ± 0.24, and 4.17 ± 0.12, respectively. The disinfectant effectiveness of BAC increased with increasing concentrations and reaction times, and 0.2% BAC demonstrated a strong disinfectant effectiveness against HCoV-OC43, as did 50, 60, and 80% EA and 70% IPA. HCoV-OC43 was rapidly (within 5 s) and completely inactivated with 10% PVP-I, similar to the case with 50, 60, and 80% EA and 70% IPA.

## DISCUSSION

The stability of HCoV and the disinfectant effectiveness against HCoV on the surface of human skin, which are important data for the prevention of contact transmission, had not been previously investigated and were unknown. This study revealed the stability and disinfectant effectiveness using our previously established evaluation model (26–28).

Our environmental stability assessment showed that the survival time of HCoV-OC43 on the surface of human skin was approximately 25 h, which was more than 2-fold longer than that of SARS-CoV-2. Although SARS-CoV-2 reportedly demonstrates higher environmental stability than influenza virus (26), our study suggests that HCoV-OC43 demonstrates even higher environmental stability. Therefore, HCoV-OC43 may have a higher contact transmission risk than SARS-CoV-2. The mechanism by which differences in environmental stability occur has not been clarified at this stage. However, a previous study on SARS-CoV-2 reported different environmental stability in each variant (29), and the environmental stability might differ depending on the spike protein. Therefore, the difference in environmental stability between SARS-CoV-2 and HCoV-OC43 might also be due to the difference in spike proteins. However, further research is needed to test this hypothesis.

Previous studies have demonstrated the efficacy of alcohol-based disinfectants against SARS-CoV-2 (30–32). In addition, our previous study showed that influenza virus and SARS-CoV-2 were completely inactivated *in vitro* with 40% EA for 5 s (27). However, the *in vitro* disinfectant effectiveness evaluation showed that HCoV-OC43 was not inactivated after reaction with 40% EA for 60 s and that the required EA concentration for HCoV-OC43 disinfection was significantly higher than that for SARS-CoV-2. Thus, HCoV-OC43 was shown to have higher EA resistance than SARS-CoV-2. Ethanol is known to be highly effective against enveloped viruses, as opposed to nonenveloped viruses (33). Nevertheless, HCoV-OC43 showed a relatively high EA resistance among the enveloped viruses compared to influenza virus and SARS-CoV-2. These findings suggest that the disinfectant effectiveness against each virus targeted for infection control should be evaluated and confirmed individually.

However, unlike the results of the *in vitro* evaluation, HCoV-OC43 on the skin surface was rapidly inactivated with 50% EA. Rubbing 52% (wt/wt) or more ethanol onto the hands for at least 15 s is a hand hygiene practice recommended by the World Health Organization (33). Therefore, the current EA-based disinfectants for hand hygiene can adequately inactivate HCoV-OC43 on the surface of human skin. HCoV-OC43 was also rapidly inactivated by 70% IPA and 10% PVP-I. While 50% or more EA-based disinfectants are effective are suitable for hand hygiene targeted against HCoV-OC43, our results suggest that 70% IPA-based disinfectants and 10% PVP-I-based disinfectants are suitable alternatives. Additionally, high concentrations of CHG and BAC, such as 1.0% CHG and 0.2% BAC, showed a relatively high disinfectant effectiveness at reaction times of 15 s or longer but were slightly less effective than 50% or more EA. Therefore, the high concentrations of CHG and BAC-based disinfectants may be used as alternatives to alcohol-based disinfectants.

This study had some limitations that should be mentioned. First, HCoV-OC43 was diluted with phosphate-buffered saline (PBS) for evaluation. Changing the solvent used for diluting the virus may affect the results of the evaluation. Second, since the HCoV-OC43 strain used in this study may have adapted during passaging in cell lines or in mice at the ATCC, the environmental stability of this strain may differ from that of primary isolates.

In conclusion, this study elucidated the stability of HCoV-OC43 on the surface of human skin and examined the disinfectant effectiveness of multiple agents against HCoV-OC43. The high stability of HCoV-OC43 on the surface of human skin may increase the risk of contact transmission. Furthermore, although HCoV-OC43 has relatively high ethanol resistance, appropriate hand hygiene practices can sufficiently reduce the contact transmission risk.

## MATERIALS AND METHODS

### Viruses and cells.

VeroE6 cells expressing TMPRSS2 (VeroE6/TMPRSS2 cells; number JCRB1819) were purchased from the Japanese Collection of Research Bioresources Cell Bank (Osaka, Japan) and cultured in Dulbecco’s modified Eagle’s medium (DMEM; Sigma-Aldrich, St. Louis, MO, USA) supplemented with 5% fetal bovine serum (FBS) and G418 (Nacalai Tesque, Kyoto, Japan) (34, 35). HCoV-OC43 (ATCC VR-1558) was purchased from the American Type Culture Collection (Manassas, VA, USA). HCoV-OC43 was cultured in Vero E6/TMPRSS2 cells and stored as a working stock at −80°C. HCoV-OC43 was concentrated and purified as follows. VeroE6/TMPRSS2 cells cultured in DMEM supplemented with 10% FBS and G418 were inoculated with HCoV-OC43 and incubated at 33°C for 7 days, and the culture medium was harvested. The harvested culture medium was centrifuged at 4°C and 3,000 rpm for 5 min to remove cell debris. After centrifugation, the supernatants were centrifuged again at 1,500 rpm for 20 min at 4°C and sterilized by passage through a 0.45-μm filter. Thereafter, virions in the supernatant were sedimented through a 20% (wt/wt) sucrose cushion in PBS with ultracentrifugation at 28,000 rpm for 2.5 h at 4°C in a Beckman SW28 rotor (27, 36).

The titer of infectious HCoV-OC43 virions was measured by the TCID_50_ assay using Vero E6/TMPRSS2 cells as reported in our previous studies (28, 37). Specifically, VeroE6/TMPRSS2 cells cultured in 96-well plates were infected with HCoV-OC43, and CPE was observed using an inverted light microscope (Olympus IX71; Olympus, Tokyo, Japan) 4 to 5 days postinfection.

### Collection of human skin specimens and preparation of the evaluation model for disinfectant effectiveness and viral stability.

Human skin was collected from forensic autopsy specimens obtained from the Department of Forensic Medicine, Kyoto Prefectural University of Medicine, within 24 h after patient death. Abdominal skin autopsy specimens from subjects aged 20 to 70 years were excised into rectangles greater than 5 by 10 cm^2^, which were used for subsequent evaluation (38). Additionally, the disinfectant effectiveness against viruses and viral stability on human skin were evaluated using a previously established human skin model (26, 27). Specifically, the subcutaneous tissue from human skin was rapidly removed, and the treated skin (mainly epidermal and dermal layers) was rinsed with PBS and placed onto a culture insert with an 8.0-μm-pore-size membrane (Corning, Corning, NY, USA). The culture inserts were placed into 12-well plates containing 1.0 mL of DMEM in each well.

### Evaluation of HCoV-OC43 stability on the surface of human skin.

The survival time of HCoV-OC43 on the skin was evaluated using our previously reported protocol (26). The same evaluation was performed on SARS-CoV-2 as a comparison. Specifically, 2 μL of HCoV-OC43 or SARS-CoV-2 (2.0 × 10^5^ TCID_50_s in 2 μL of PBS) was applied to skin samples and incubated at 25°C under a relative humidity of 45 to 55% for 0 to 24 h. After incubation, residual viruses on the skin were recovered with 1 mL of DMEM and then titrated. The survival time was defined as the time until the virus on the skin was not detected. The titer detection limit of each virus was 3.2 × 10^0^ TCID_50_s.

### *In vitro* evaluation of disinfectant effectiveness against HCoV-OC43.

To evaluate target disinfectants, 80, 60, 50, 40, and 20% (wt/wt) EA (Nacalai Tesque), 70% (wt/wt) IPA (Nacalai Tesque), 0.2 and 1.0% (wt/vol) CHG (Fujifilm Wako Pure Chemical Corporation, Osaka, Japan), 0.05 and 0.2% (wt/vol) BAC (Fujifilm Wako Pure Chemical Corporation), and 10% (wt/wt) PVP-I (Sigma-Aldrich) were used. CHG and BAC are disinfectants currently used for hand hygiene, with CHG and BAC mainly used at 0.2 to 1.0% and 0.05 to 0.2%, respectively. Dermal toxicity and appropriate concentrations for the skin have been reported in previous studies, and the concentrations of CHG and BAC used in this study were based on these reports (39–44).

The *in vitro* evaluation protocol was based on a previous study (27). Briefly, HCoV-OC43 was mixed with PBS to adjust the viral titer to 5.0 × 10^7^ TCID_50_s/mL before exposure to each disinfectant. Next, 36 μL of each disinfectant was added to 4 μL of the virus mixture (2.0 × 10^5^ TCID_50_s in 4 μL of the virus mixture) and incubated at 25°C for 5, 15, and 60 s. After incubation, 360 μL of soybean-casein digest broth with lecithin and polysorbate 80 (SCLDP; Nihon Pharmaceutical, Tokyo, Japan) and 400 mg of nonpolar polystyrene adsorbent (Bio-Beads SM-2 resin; Bio-Rad, Hercules, CA, USA) were added to each tube to neutralize the disinfectant and then mixed at 25°C for 30 min. Finally, 1,600 μL of DMEM was added and the remaining viral titers in final solutions were measured. During the evaluation of PVP-I, 0.5% sodium thiosulfate (Nacalai Tesque) was used instead of SCLDP. The titer detection limit of HCoV-OC43 was 6.3 × 10^0^ TCID_50_s. For evaluation of disinfectant effectiveness, the log reduction was calculated using the viral titer when PBS was added as a control, instead of each disinfectant. Three independent experiments were conducted, and the results were expressed as means ± standard errors.

Moreover, the effect of EA on HCoV-OC43 was evaluated in detail. The same evaluation was performed on SARS-CoV-2 as a comparison. To evaluate EA effectiveness, we used 80, 60, 55, 50, 45, 40, 35, 30, 25, and 20% (wt/wt) EA. Briefly, HCoV-OC43 and SARS-CoV-2 were mixed with PBS to adjust the viral titer to 5.0 × 10^7^ TCID_50_s/mL before exposure to each disinfectant. Next, 36 μL of each disinfectant was added to 4 μL of the virus mixture (2.0 × 10^5^ TCID_50_s in 4 μL of virus mixture) and incubated at 25°C for 15 s. After incubation, 360 μL of SCLDP and 400 mg of nonpolar polystyrene adsorbent were added to each tube to neutralize the disinfectant, and then the sample was mixed at 25°C for 30 min. Finally, 1,600 μL of DMEM was added, and the remaining viral titers in final solutions were measured. The titer detection limits of HCoV-OC43 and SARS-CoV-2 were 6.3 × 10^0^ TCID_50_s.

### Evaluation of disinfectant effectiveness against HCoV-OC43 on human skin.

The same disinfectants for the *in vitro* assay were evaluated on the surface of human skin. The evaluation protocol was based on a previous study (27). Before exposure to each disinfectant, HCoV-OC43 was mixed with PBS to adjust the virus titer to 1.0 × 10^8^ TCID_50_s/mL. The viral mixture (2.0 × 10^5^ TCID_50_s in 2 μL of virus mixture) was applied to the epidermis of the skin, and the samples were incubated for 10 min at 25°C under a relative humidity of 45 to 55% to dry the viral mixture on the skin completely. Next, 40 μL of each disinfectant was applied to the skin, incubated for 5, 15, and 60 s, and then air dried for 2 to 3 min. After drying, the remaining viruses on the skin were recovered using 500 μL of neutralizing medium (SCLDP or 0.5% sodium thiosulfate), and the neutralizing medium and 400 mg of nonpolar polystyrene adsorbent were then mixed at 25°C for 30 min. Finally, 1,500 μL of DMEM was added and then titrated. The titer detection limit of HCoV-OC43 was 6.3 × 10^0^ TCID_50_s. For evaluation of disinfectant effectiveness, the log reduction was calculated using the viral titer when PBS was added as a control, instead of each disinfectant. Three independent experiments were conducted, and the results were expressed as means ± standard errors.

### Ethical considerations.

The protocol for this study was approved by the Ethics Committee of Kyoto Prefectural University of Medicine (ERB-C-1593). Written informed consent was obtained from the bereaved families of the study subjects.

### Statistical analysis.

Data were analyzed using GraphPad Prism 7 software (GraphPad, La Jolla, CA, USA). For the evaluation of EA effectiveness, EA concentration was defined as the explanatory variable (*x* axis) and the log viral titer as the explained variable (*y* axis). A nonlinear regression analysis was performed using a four-parameter logistic model (45). The EA concentration required to exceed a log reduction of 3.5 in a 15-s reaction with a disinfectant was defined as the *x* value when the *y* value of the regression curve was 1.3. For the evaluation of the survival time and half-life, the elapsed time was defined as the explanatory variable (*x* axis) and the log viral titer of HCoV-OC43 and SARS-CoV-2 as the explained variables (*y* axis). A least-squares linear regression analysis was performed using the logarithmic link function for each virus to create a regression curve. The survival time of each virus was defined as the *x* value when the *y* value of the regression curve was 0.5. The half-life was calculated from the slope of each regression curve when the residual viral titers on the skin were 2.0 logTCID_50_s and 3.0 logTCID_50_s.

### Data availability.

All data included in this study are available from the corresponding author on request.
