# Supplementary material for: Evaluation of Environmental Stability and Disinfectant Effectiveness for Human Coronavirus OC43 on Human Skin Surface
Source: Microbiol Spectr. 2023 Feb 22;11(2):e02381-22. doi: 10.1128/spectrum.02381-22 (PMC10100891; doi:10.1128/spectrum.02381-22)
Supplement: Supplemental file 1 — Fig. S1. Download spectrum.02381-22-s0001.pdf, PDF file, 0.1 MB [file spectrum.02381-22-s0001.pdf]

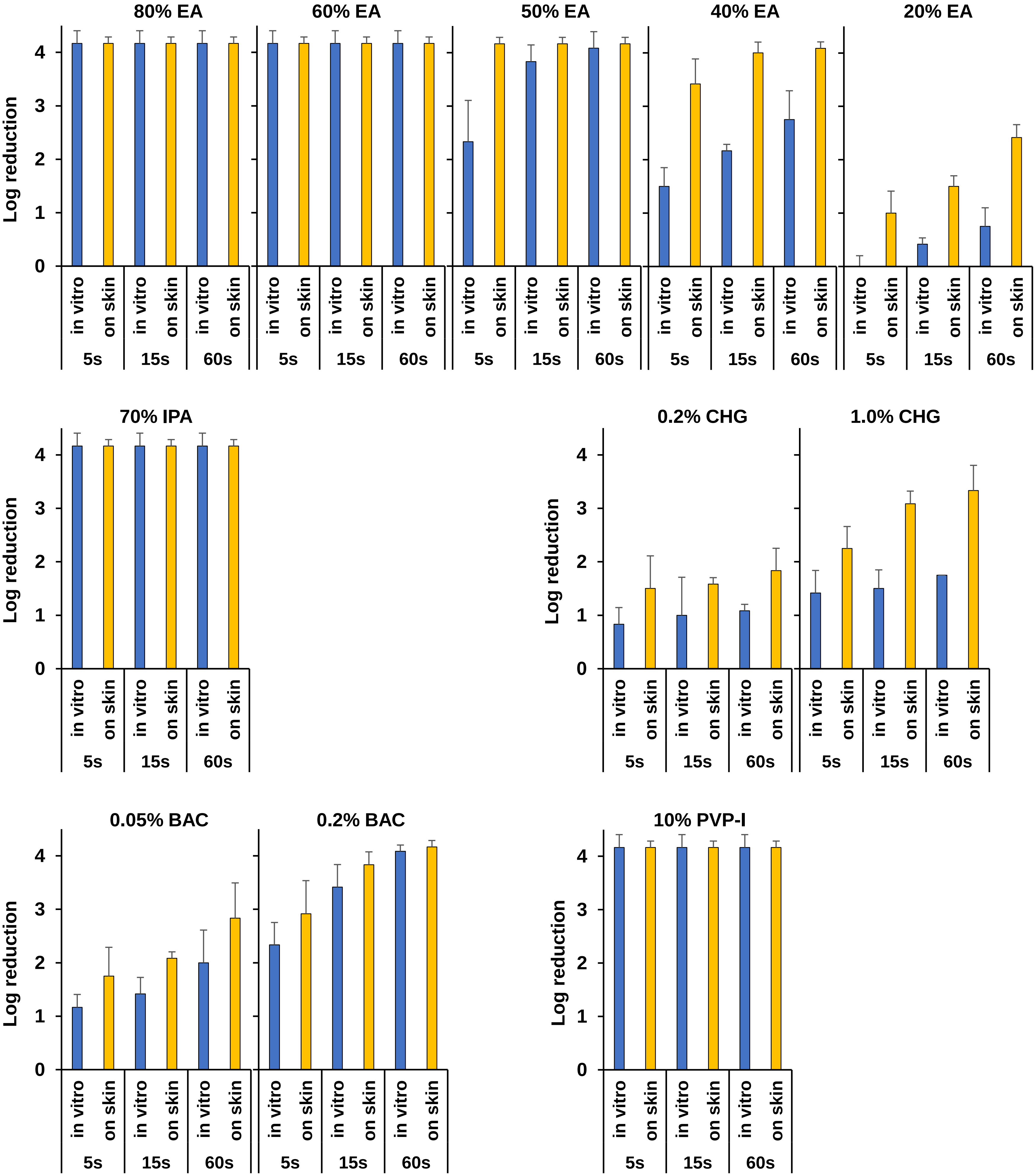

Supplementary Figure S1. Evaluation of disinfectant effectiveness against HCoV-OC43 on the surface of human skin and *in vitro* disinfectant effectiveness evaluation.

EA, ethanol; IPA, isopropanol; CHG, chlorhexidine gluconate; BAC, benzalkonium chloride; PVP-I, povidone iodine; HCoV-OC43, human coronavirus-OC43. The log reduction value was calculated to evaluate the disinfectant effectiveness under each condition and was expressed as mean ± standard error.
